# Operating characteristics of the factor flow networks in rural areas: A case study of a typical industrial town in China

**DOI:** 10.1371/journal.pone.0283232

**Published:** 2023-03-16

**Authors:** Zhi Li

**Affiliations:** 1 School of Geographical Sciences, Hebei Normal University, Shijiazhuang, Hebei, China; 2 Hebei Key Laboratory of Environmental Change and Ecological Construction, Shijiazhuang, Hebei, China; 3 Hebei Technology Innovation Center for Remote Sensing Identification of Environmental Change, Shijiazhuang, Hebei, China; 4 Hebei Key Research Institute of Humanities and Social Sciences at Universities “GeoComputation and Planning Center of Hebei Normal University”, Shijiazhuang, Hebei, China; Shenzhen University, CHINA

## Abstract

The networks of factor flows in rural areas are the main support for rural revitalization, which has become one of the research trends in rural geography. Taking a typical industrial town in China as an example, the study explored the operating characteristics of rural factor flow networks and the relations of multi-factor flows based on the social survey method and fine-grained flows data. Results showed that population flows, capital flows and policy flows increased significantly in rural areas. Thereinto, population flows, especially labor flows, mainly ran into the townships and industrial cluster villages, so did capital inflows and outflows, while policy flows ran around the township. The villages with dense population and capital flows formed the "central villages", which had exceeded the township in the two flow networks. Policy flows and capital flows played a guiding role in population flows, so did the policy flows on the capital flows. Meanwhile, the population flows and the capital flows could reinforce each other. In conclusion, a multi-center structure network with the separation of economic center and administrative center had been formed in rural areas. And there was a close interaction between these factor flows. Furthermore, the theoretical model of town-village symbiotic network was constructed.

## Introduction

Since the 1990s, globalization and information technology has developed rapidly, and the global flows of a large number of physical and virtual factors have increased significantly, which leads to the fact that mobility has become an important field in geography and formed a "new flow paradigm" that emphasized dynamic research [1–3]. With the expansion and distribution of modern transportation and information infrastructure, new economic business and high-quality public services towards grassroots villages, the flows of population, capital, information, traffic and service in rural areas have grown vigorously, and together, they formed a closely interaction and open rural network [4–7]. In the factor flows network, the connection between urban and rural areas is increasingly enhanced, and the rural socio-economic operation has greater location elasticity, indicating that rural areas show obvious mobility. The fact promotes the shift from static local research to dynamic flow networks research, and the shift of the theoretical basis from "space of places" (with location as the core attribute) to "space of flows" (with interaction as the core attribute) [8–10]. Under the guidance of “space of flows" theory, a research trend of "flow turning" raised in rural geography [11]. That is, the theoretical and empirical researches on rural network of factor flows are increasing, and the research objects include information technology change and rural transformation [12–14], mobility practice and significance [15,16], mobility inequality [17,18], and the impact of mobility on rural localities and society [9,19–21]. Thereinto, analyzing the operation and structure of factor flows network is the research focus of rural geography.

Since 21st century, the big data and data science developed vigorously, providing a number of relational data and methods that can directly measure traffic flows, information flows, population flows and knowledge flows for geographers, and promoting the study of regional network of factor flows [22–24], as well as the new methods such as complex network analysis [25,26], big data models [27], graph theory [28] and flow maps analysis [29–31]. Factually, most of the related studies have focused on more mobile, data-rich and accessible urban areas, while few on the rural areas [32–34]. At present, the research objects of rural network of factor flows were mainly the network of population flows [35–39], traffic flows [40,41], tourism flows [42,43] and information flows [12,44]; research contents included network deconstruction, dynamic evolution, spatio-temporal pattern, influencing factors and optimization strategies; research methods involved in gravity model, field strength model, potential model, breaking point analysis and qualitative study [45–49]. However, these researches mainly focused on the city scale where statistical data are abundant and easily available [50], or the village scale where information flows are dense, such as Taobao village and tourist village [51,52], whereas lacking in the systematic analysis of the town scale. In the meanwhile, the researches of multi-factor flows were far less than that of single-factor flows. Also, the methods were still mainly static relational data analysis, rather than dynamic data analysis. These deficiencies could not well meet the theoretical and practical needs of rural revitalization in the new era.

China is a developing country with large population and little arable land, and rural development is facing the dilemma of low per capita resources and the large gap between urban and rural areas. The National 14th Five-Year Plan clearly stated that "fully implement the rural revitalization strategy and encourage more production factors to flow towards rural areas to comprehensively improve the efficiency of rural operations". Moreover, the operating efficiently of rural factor flow networks plays a crucial role in promote rural revitalization, which has currently become a scientific problem to be solved for rural geographers. Therefore, taking a typical industrial town in traditional agricultural areas in Hebei Province, China, as an example, this article aimed to analyze the operating characteristics of the factor flow networks and the relationship of multi-factor flows based on the theory of “space of flows”, and to explore the influencing elements and theoretical model of factor flow networks in Chinese traditional rural areas, in order to provide scientific references for rural revitalization.

## Materials and methods

### Study area

Traditional agricultural area is one of the most important research areas of rural geography, as well as the main practical object of rural revitalization. Thereinto, the south of Hebei Province is a typical representative with underdeveloped economy, while it plays a crucial role in agricultural production. Because of rural settlements overloaded in number and density, the per cultivated area is less than 0.12 hm^2^, implying the prominent contradiction between man and land, and insufficiency of agricultural development in supporting rural revitalization. Therefore, rural revitalization in traditional agricultural areas needs theoretical and methodological guidance.

The Jiajiakou town, located in Ningjin County, Hebei Province, is a typical industrial town in the traditional agricultural areas. The town covers an area of 94.3 km^2^, with 21 administrative villages under its jurisdiction. The town government is located in Jiajiakou village (township). At the end of 2020, the total number of households in Jiajiakou Town has reached 18,869, with a registered population of 59,192 and a permanent resident population of 54,622. Provincial Road 393 runs through the town from east to west, and Highway 20 establishes an entrance and exit in the town at the intersection with Provincial Road 393. Thus, regional transportation is very convenient. Since the 1990s, Jiajiakou town has developed gradually from a traditional grain-producing region into a national production base of wire and cable, and has been awarded the titles as "Famous Town of Chinese Economy and Culture" and "Important Town of Chinese Wire and Cable Production". There are 1,040 private enterprises and 10,833 workers in the town, creating an output value of 22.7 billion yuan and a profit of 2.4 billion yuan annually. Of the 1040 industrial enterprises, 45 have fixed assets over 20 million yuan, which are all wire and cable enterprises.

In short, Jiajiakou town has realized the transformation and upgrading of rural industries, taking into account the task of grain production, and promoting the sustainable development of rural economy and society. Currently, the rural development level ranks the first in the county, becoming one of the economically-developed industrial towns in China, and is representative and demonstrative in Chinese traditional agricultural areas. It is rated as "China’s Rural Revitalization Demonstration Area" and "China’s economic and cultural Town".

Therefore, this paper took Jiajiakou town as an example to study the operation characteristics of rural factor flow networks, and to provide certain reference for rural revitalization in traditional agricultural areas. In addition, the Yaotai 1 village, the Yaotai 2 village and the Yaotai 3 village are combined into a natural village named the Yaotai village, since they are adjacent to each other in geographical space and are highly integrated in economy, society and culture. Similarly, the East Huangerying village and West Huangerying village were merged into a natural village named Huangerying village. This study taked the 18 villages (township) that make up the Jiajiakou town as the research objects. Thereinto, Jiajiakou village/township is the administrative center in the town.

### Data source and methods

The data in this study involved three parts: population flows, capital flows, and policy flows. (1) The registration forms of primary school students in 2020 were collected from Jiajiakou town government. (2) The sample size of the survey was calculated based on the stratified sampling method and was composed of 5% of all households in each village. The data on income and consumption capital flows of rural residents in 2020 were collected by questionnaires and semi-structured interviews. A detailed questionnaire containing 60 items was designed and the items were classified into three categories, including the flows of households’ income, the flows of consumption, and the infrastructure and public services. A total of 1000 questionnaires were issued in the participatory survey and 987 were collected, among which 951 were valid. (3) The data of administration district referred to “Ningjin County statistical yearbook” [53–55] were provided by Ningjin County Statistics Bureau. Data of rural policy flows were processed and obtained by administration district data.

This paper combined qualitative and quantitative methods to study the operating characteristics of the factor flow networks in rural areas. Through the social survey and spatial analysis method, the operating process of population flow network, capital flow network and policy flow network in rural areas were revealed, as well as the relationship of multi-factor flows in rural areas. The social survey method was used to explore the influencing elements by analyzing the rural endowments and the regional environment. Combined with social investigation and qualitative analysis, this paper further constructed the theoretical model of town-village symbiotic network.

## Results

### Operating characteristics of rural population flow network

Before the 1990s, the daily activities of the rural residents were mainly around agricultural production. Market transactions and social communication were concentrated insides villages and towns, without frequent movements of population [56]. In the 1990s, with the rapid development of industrialization and urbanization in China, the rural population mainly flowed out to Jiajiakou, Huangerying, Yaotai and Xiaoliu villages with a large number of industrial enterprises. Since the 21st century, the non-agricultural industries in these villages continued to develop, and the rural infrastructure and public service resources were speedily integrated or gathered into the township and these villages [56]. These villages were able not only to provide sufficient non-agricultural jobs for villagers, but also to act as the important supply bases of goods and public services for the surrounding villages, contributing to a significant increase in rural population flows.

**Fig 1:**
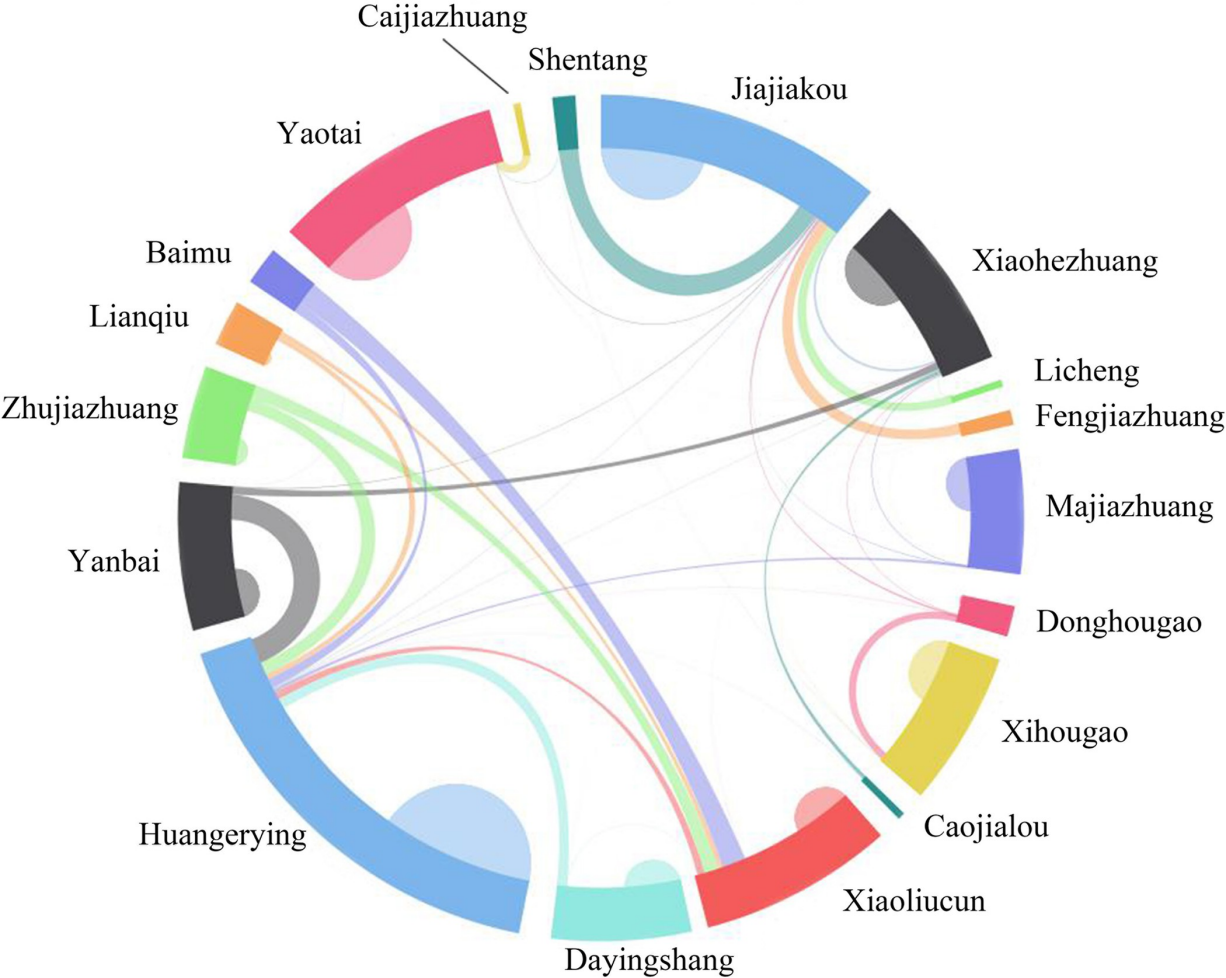
Flows matrix of the primary school students in Jiajiakou town.

Based on the information about the residence and domicile places of all students in Jiajiakou town, the spatial flows matrix of rural students was obtained (Table 1, Fig 1). These students mainly flowed to Jiajiakou, Huangerying, Yaotai, Xiaohezhuang, and Xiaoliu villages for two reasons. On the one hand, to cope with the loss of rural population and promote the optimal allocation of educational resources, Ningjin county has started to withdraw and merge primary schools according to the principle of proximity. Specifically, the primary schools in the villages with less population have been merged into those in the surrounding township or Jiajiakou, Huangerying, Yaotai, Xiaohezhuang, and Xiaoliu villages. On the other hand, a large number of processing and manufacturing enterprises have been established in Jiajiakou, Huangerying, Yaotai and Xiaohezhuang, which attracts numerous labor forces from surrounding villages, and they decided to live and educate their children in the place where they work. At present, commodity, education and medical resources from Jiajiakou, Huangerying, and Yaotai villages have effectively radiated to the surrounding villages, and these villages have become the agglomeration centers of population flow network. Notably, Huangerying village was more attractive to the surrounding population compared to Jiajiakou village(township).

**Table 1:** Spatial flows matrix of primary school students in Jiajiakou town.

Inflow site Outflow site	Jiajiakou	Xiaohezhuang	Licheng	Fengjiazhuang	Majiazhuang	Donghougao	Xihougao	Caojialou	Xiaoliu	Dayingshang	Huangerying	Yanbai	Zhujiazhuang	Lianqiu	Baimu	Yaotai	Caijiazhuang	Shentang
Jiajiakou	**515**	**9**	0	0	0	0	0	0	0	0	**4**	0	0	0	0	**3**	0	0
Xiaohezhuang	**1**	**346**	0	0	0	0	0	0	0	0	**1**	0	0	0	0	0	0	0
Licheng	**43**	**3**	0	0	**1**	0	0	0	0	0	0	0	0	0	0	**1**	0	0
Fengjiazhuang	**52**	0	0	0	0	0	0	0	0	0	**1**	0	0	0	0	**1**	0	0
Majiazhuang	**5**	**4**	0	0	**267**	0	0	0	0	0	**14**	0	0	0	0	0	0	0
Donghougao	**10**	**3**	0	0	0	**49**	**35**	0	0	0	**2**	0	0	0	0	0	0	0
Xihougao	**1**	**2**	0	0	0	0	**317**	0	0	0	0	0	0	0	0	0	0	0
Caojialou	0	**20**	0	0	0	0	0	0	0	0	**1**	0	0	0	0	0	0	0
Xiaoliu	0	**1**	0	0	0	0	0	0	**293**	0	**37**	0	0	0	0	0	0	0
Dayingshang	**1**	**1**	0	0	0	0	0	0	**2**	**282**	**51**	0	0	0	0	0	0	0
Huangerying	0	**1**	0	0	0	0	0	0	0	0	**760**	0	0	0	0	0	0	0
Yanbai	**4**	**36**	0	0	0	0	0	0	0	0	**125**	**241**	0	0	0	0	0	0
Zhujiazhuang	0	0	0	0	**1**	0	0	0	**70**	0	**63**	0	**138**	0	0	0	0	0
Lianqiu	0	0	0	0	0	0	0	0	**25**	0	**26**	0	0	**71**	0	0	0	0
Baimu	0	0	0	0	0	0	0	0	**116**	0	**48**	1	0	0	0	0	0	0
Yaotai	**2**	**1**	0	0	0	0	0	0	0	0	0	0	0	0	0	**489**	0	0
Caijiazhuang	0	0	0	0	0	0	0	0	0	0	**1**	0	0	0	0	**41**	0	0
Shentang	**96**	0	0	0	0	0	**1**	0	0	0	0	0	0	0	0	**2**	0	0

Factually, in the population flow network, most rural population flowing was due to employment, thus forming the labor flow networks, which could more objectively reflect the situation of rural transformation and development. Before industrialization, rural labor force was mainly engaged in agricultural production or handicraft activities, mainly flowing inside the village. With the development of industrialization and urbanization, rural labor force generally turned to non-agricultural production activities. Part of the labor force flowed out to the urban areas, and part flowed to the villages where industrial enterprises gathered, such as Huangerying village, Jiajiakou village and Yaotai village. The mobility of rural labor force increased significantly. Therefore, the operation of rural labor flow network was closely related to economic production. The inflow nodes were the settlements with abundant non-agricultural jobs, and the outflow nodes were the general villages.

### Operating characteristics of rural capital flow network

Before the 1990s, the income of rural residents was mainly from the family agricultural production and their daily consumption was insides their own villages, leading to the weak liquidity in rural areas [56]. Since the 1990s, Jiajiakou, Huangerying, and Xiaohezhuang villages had established dozens of wire and cable processing plants, producing a lot of capital flows, and many labors flowed into the manufacturing enterprises. As a result, non-agricultural income became the main livelihood source of households, and a large number of capital flows grew constantly.

**Fig 2:**
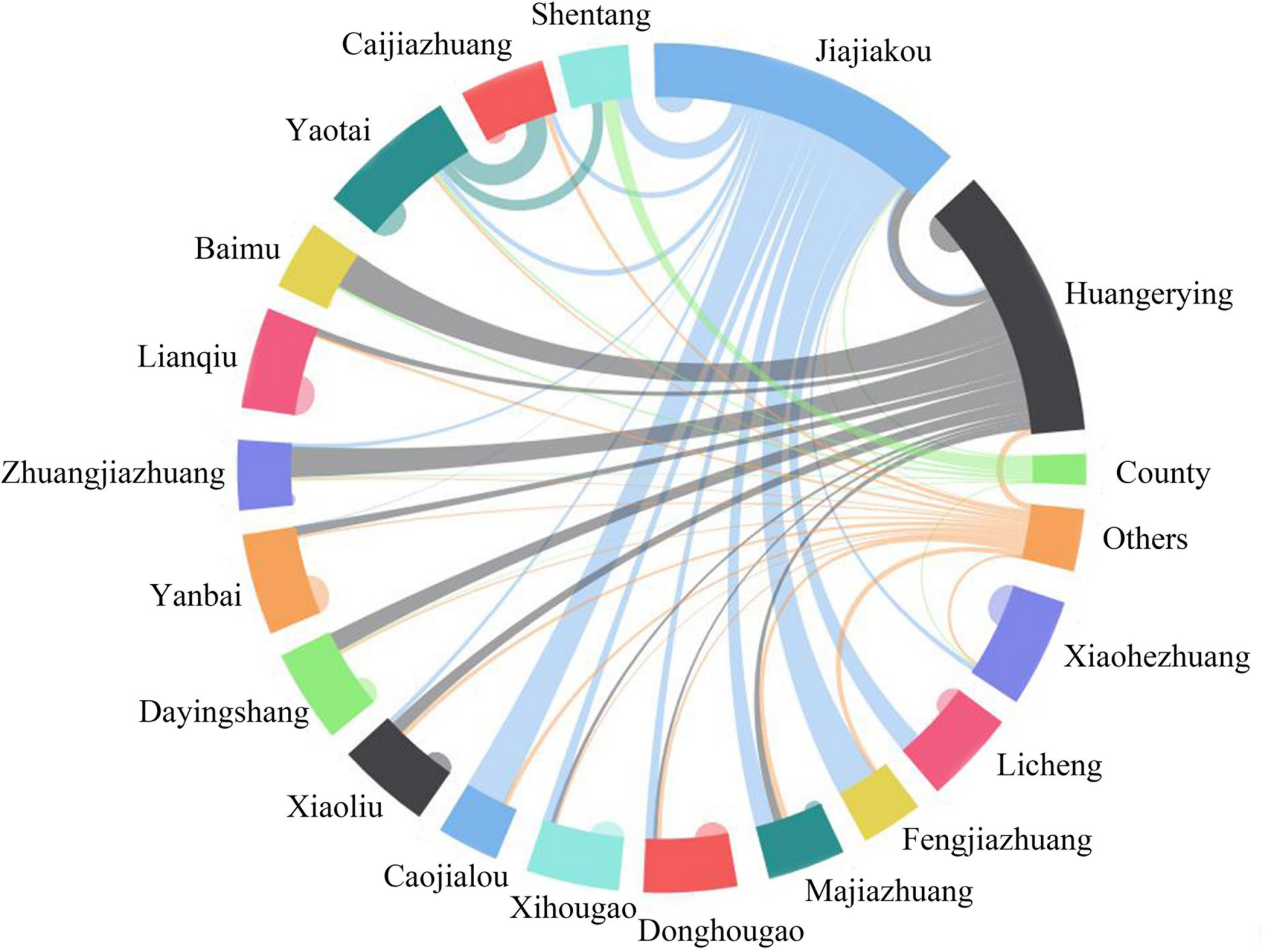
Proportion of income source sites in Jiajiakou town.

The proportion of income source sites of the households from Jiajiakou town in 2020 was surveyed and presented in Table 2 and Fig 2. On average, 36% of households obtained their main income from their own villages, 35% obtained from Huangerying and Yaotai villages, and 21% obtained from the township, respectively. The Huangerying village was the one with the highest proportion of households whose income was from their own villages (82%), while Fengjiazhuang (10%) and Caojialou (10%) showed the lowest proportion. The results indicated that Jiajiakou, Huangerying and Yaotai villages have become the main income source sites for the villagers, which was the places for the concentration of capital flows.

**Table 2:** Proportion of income source sites in Jiajiakou town (%).

Income source sites Villages	Inside village	Huangerying or Yaotai village	Township	County	Others^a^
**Jiajiakou**	74	21	-	3	2
**Xiaohezhuang**	82	0	11	3	5
**Licheng**	60	0	40	0	0
**Fengjiazhuang**	10	0	80	0	10
**Majiazhuang**	33	17	38	0	13
**Donghougao**	64	8	20	0	8
**Xihougao**	65	9	22	0	4
**Caojialou**	10	0	80	0	10
**Xiaoliu**	55	26	10	0	10
**Dayiingshang**	55	38	0	3	5
**Huangerying**	82	-	6	0	12
**Yanbai**	74	19	3	0	4
**Zhujiazhuang**	23	60	10	3	3
**Lianqiu**	77	15	0	0	8
**Baimu**	21	71	0	7	0
**Yaotai**	77	-	11	6	6
**Caijiazhuang**	40	40	10	0	10
**Shentang**	20	20	30	30	0
**Average**	36	35	21	2	6

^a^ Contains other cities, counties, towns, and Internet etc.

**Fig 3:**
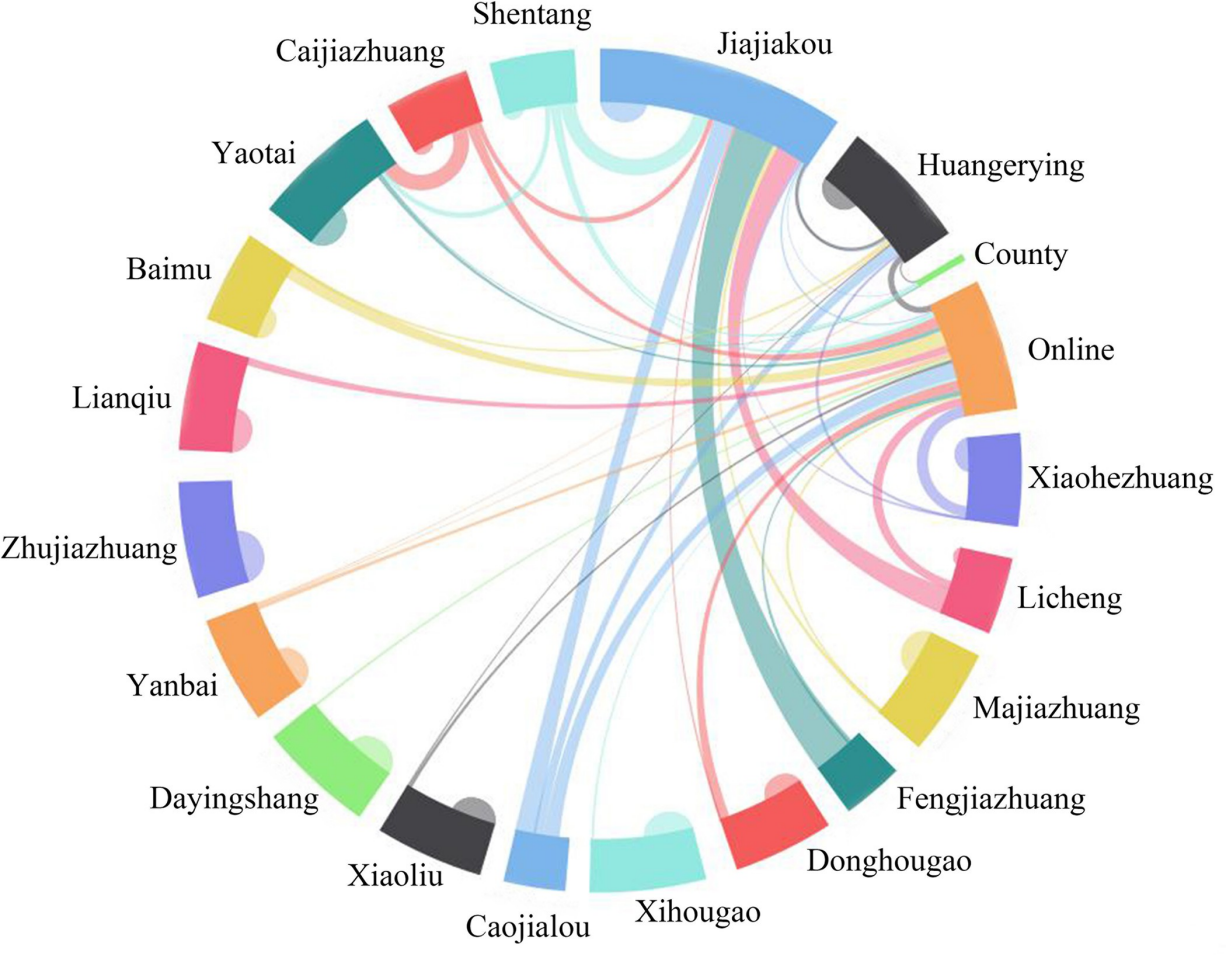
Proportion of daily living consumption in Jiajiakou town.

**Fig 4:**
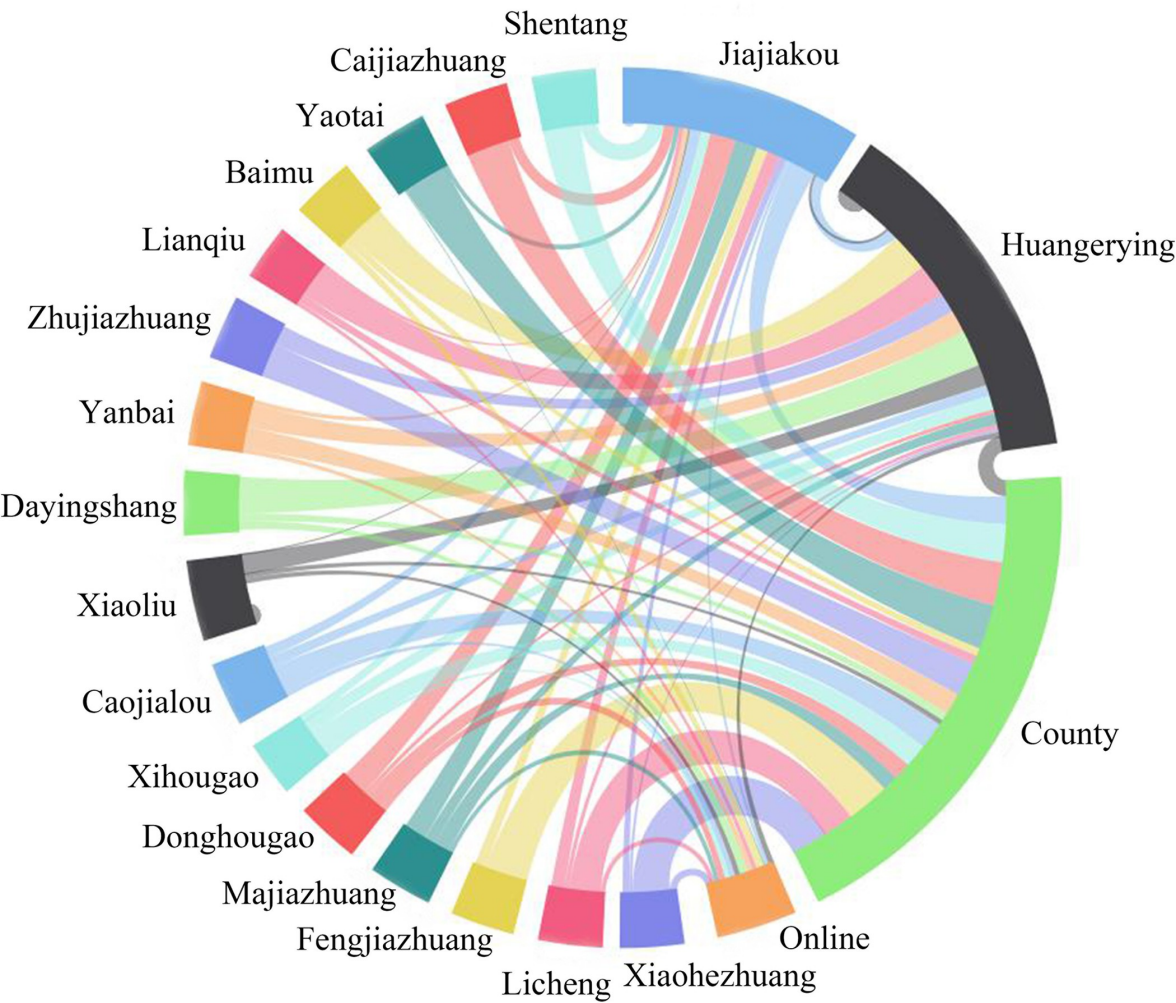
Proportion of large-item consumption in Jiajiakou town.

The data of daily living and large-item consumption sites of households in Jiajiakou town were obtained through related surveys (Table 3, Figs 3 and 4). The results showed that the living consumption fund flowed mainly into four directions, including their own villages (50% households), Huangerying and Yaotai villages(21%), Jiajiakou township (17%) and online consumption (10%);. Large-item consumption fund flowed into the county (44% households), central villages (31%), township (14%), the network (8%) and their own villages (3%). Though there are obvious differences in flow direction between daily living and large-item consumption of rural residents, Jiajiakou, Huangerying and Yaotai villages have become the main sites of capital inflow in the town.

**Table 3:** Proportion of daily/large item consumption sites in Jiajiakou town (%).

Consumption sites Villages	Inside village	Huangerying or Yaotai village	Township	County	Online^a^	Others^b^
**Jiajiakou**	90/23	3/20	-	2/50	3/7	2/0
**Xiaohezhuang**	66/0	5/5	5/13	0/68	21/13	3/0
**Licheng**	33/0	0/7	53/27	0/60	13/7	0/0
**Fengjiazhuang**	10/0	0/0	80/20	0/80	10/0	0/0
**Majiazhuang**	88/0	0/21	8/42	0/29	4/8	0/0
**Donghougao**	76/0	0/8	4/48	0/32	20/12	0/0
**Xihougao**	96/0	0/26	0/17	0/43	4/11	0/2
**Caojialou**	10/0	20/20	40/20	0/50	30/10	0/0
**Xiaoliu**	87/39	3/39	0/3	0/10	6/10	3/0
**Dayingshang**	95/3	0/68	0/0	0/15	5/15	0/0
**Huangerying**	79/56	-	6/4	3/29	12/10	0/1
**Yanbai**	84/0	1/42	0/9	1/41	9/9	4/0
**Zhujiazhuang**	97/0	2/37	0/0	0/63	2/0	0/0
**Lianqiu**	85/0	0/62	0/8	0/15	15/8	0/8
**Baimu**	64/0	7/71	0/0	0/14	29/14	0/0
**Yaotai**	89/6	-	0/9	2/83	9/2	0/0
**Caijiazhuang**	40/0	30/0	10/20	0/80	20/0	0/0
**Shentang**	40/0	10/0	30/30	10/70	10/0	0/0
**Average**	50/3	21/31	17/14	1/44	10/8	1/0

^a^ Shopping online, such as Taobao.com, JD.com, and Amazon.cn.

^b^ Contains other cities, counties, and towns etc.

The number before “/” refers to the proportion of daily consumption, and the number after “/” refers to the proportion of large item consumption.

In a word, rural capital flows showed obvious cluster characteristics, and the capital inflow and outflow were concentrated in Jiajiakou, Huangerying and Yaotai villages.

### Operating characteristics of rural policy flow network

Rural policy flows include administrative order communication, examination and approval, supervision activities and feedback of management, which were implemented and accomplished by the township government and the relevant functional departments Therefore, The operation of policy flows is around the station of township government and the relevant functional departments (administrative center) rather than among general villages. To be specific, policy flows such as administrative orders and supervision activities flowed from Jiajiakou township to all villages, while administrative examination and approval, and management feedback flowed from each village to Jiajiakou township, which showed that the rural policy flow network was with a single center structure.

In conclusion, according to the quantity and direction of rural population flows and capital flows, Huangerying and Yaotai could be regarded as economic central villages. Similarly, Jiajiakou, as the administrative township, was the center of rural policy flow network. And they were the three central nodes of factor flow networks in rural areas.

### Relationship of rural multi-factor flows

**Fig 5:**
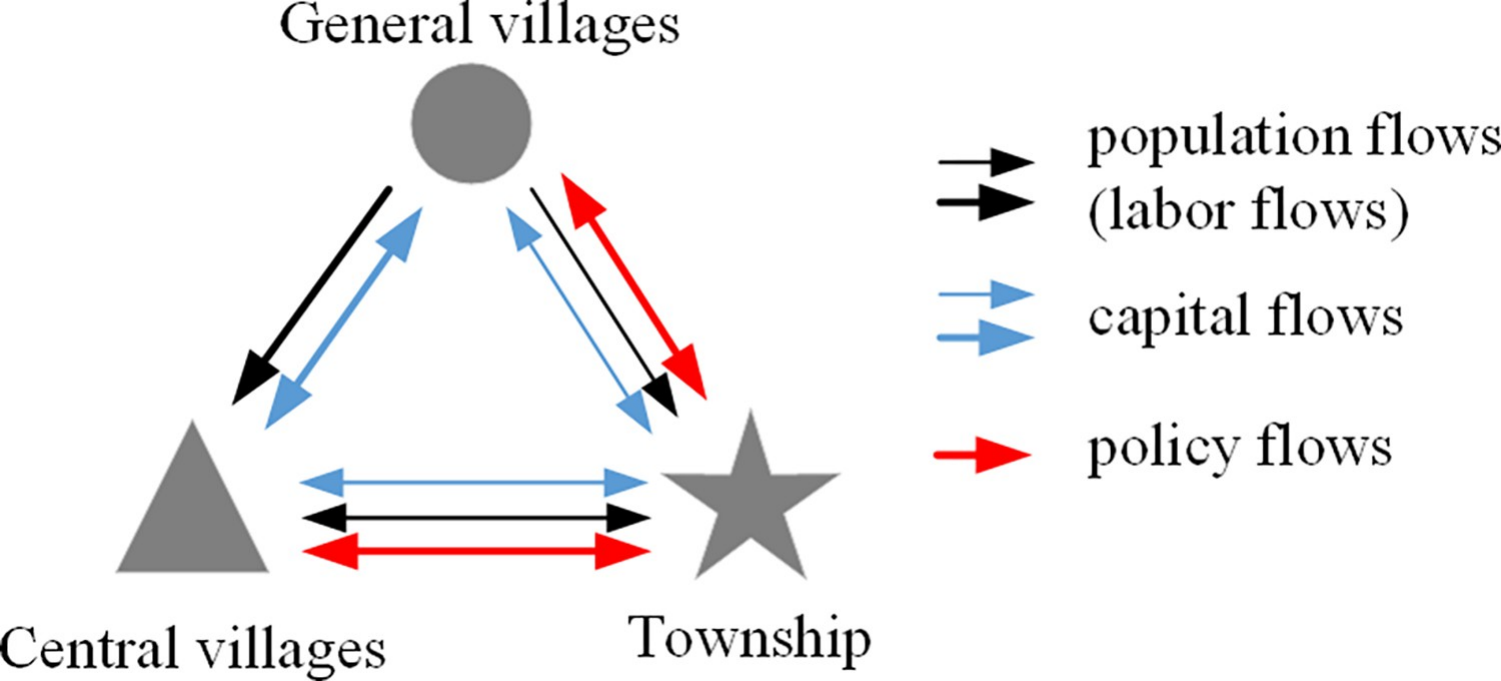
Relationship of multi-factor flows in Jiajiakou town. The width of the arrows is proportional to the volume or frequency of the flows.

Rural policy flows ran around the administrative township. In China, public service resources such as education, medical care and transportation were mainly allocated from top to bottom according to the administrative levels, and the township was the key node of basic commodities and services supply in rural areas. Rural residents need access to township in order to obtain necessary administrative approvals, public services and goods, which promoted the flowing of population and capital into the township. Therefore, policy flows had a guiding effect on the population flows and capital flows, making the township becoming the central node of the rural factor flow networks (Fig 5).

There were certain similarities in the trajectories of population flows and capital flows in rural areas, and they both were mainly around the economic and administrative centers, and the quantity of the former exceeded that of the latter. Due to the high industrialization in Huangerying and Yaotai villages, more non-agricultural jobs were created compared with the township, which were the main income sources of households and attracted a large number of population and capital inflow from the surrounding villages. With the agglomeration of industry and capital, economic central villages developed and improved the living consumption and public service functions, and then became the central nodes of the consumption flow networks of rural residents, which further enhanced the attraction of the rural population flows. This suggested that population flows and capital flows reinforced each other, and capital flows had a guiding effect on population flows, while there was certain competition between capital flows and policy flows.

In short, rural production activities and household livelihood depended on the economic centers, and rural daily life and public services depended on the administrative center. The two centers were separated in rural areas, thus forming the rural factor flow networks with multi-center structure. Moreover, the factor flows of the central villages have exceeded those of the township, which indicated that capital flows were more competitive than policy flows for attracting population flows, and the comprehensive competitiveness of the central villages has exceeded that of the township in rural areas.

## Discussion

In the operating process of rural factor flow networks, the multi-center structure had obvious advantages than the single-center structure. On the one hand, there was a cooperative relationship between the economic center and the administrative center. Because the comprehensive carrying capacity of the central town was limited. The separation of economic and administrative centers helped to reduce the carrying capacity and pressure of resources and environment in the township, to improve the accessibility of employment, goods and public services in ordinary villages, and to improve the stability of network operation. On the other hand, there was a competition relationship between economic and administrative centers for population and capital flows. Moderate competition could stimulate the vitality of rural development and improve the efficiency of regional factor mobility in rural areas, but excessive or malicious competition should be prevented.

### Related driving elements of rural factor flow networks

In the operating process of rural factor flow networks, the multi-center characteristics showed that rural units played different roles in the network, which was determined by rural endowments [57]. Rural endowments referred to the functional characteristics of the type, quantity, quality and their combination, including economic endowments (the collection of population, capital, transportation, location, and energy) [58], innovation endowments (the collection of knowledge, technology, information and talents) [47] and land endowments (the collection of Topography, landform, water and soil conditions) [59]. Due to the difference and complementarity between rural endowments, the potential energy difference was generated among rural units, which created the prerequisite for factor flowing. And then, the factor flowing further affected the structural characteristics of the factor flow networks. For example, since the 1990s, non-agricultural industries, infrastructure and public services in Jiajiakou, Huangerying and Yaotai villages had developed faster than other villages, which expanded the differences in endowments between villages. Meanwhile, economic activities required a series of production factors to gather in the place. Therefore, the rural population, capital, and organization flowed into Huangerying, Jiajiakou, Yaotai villages in large quantities, which changed the operating track of the rural factor flows, reconstructed the rural factor flow networks, and formed a multi-center structure of network.

The rural factor flow networks were an open and dynamic system, which was constantly transmitting or exchanging material, information and energy with the regional environment. Natural environment was a relatively stable geographical condition, which had few effect on the change of rural factor flows in short time. Humanistic environment was a dynamic social condition, including economic, social, and institutional environment, which regulated the operation of rural factor flow networks.

First, the development of rural industry in the central villages and township drove the upstream and downstream industrial chain, and created a large number of jobs, which attracted a large number of production and living factors flowing into. Therefore, the non-agricultural economic environment could promote the flowing of rural factors. Second, regional social environment still had relatively obvious characteristics of traditional agricultural society, such as the satisfaction with current situation, cultural conservatism, and acquaintance relationship orientation, which constrained rural population and capital flowing. Third, institutional environment could regulate the flowing trajectory of multiple factors in rural areas, and then affect the operation of rural factor flow networks. For example, since 1992, rural development policies in China had mainly experienced the reform of socialist market economy system, household registration and land system, new rural construction, new urbanization and rural revitalization. These policy reforms had removed institutional barriers to the factor flowing, significantly activated the inter-regional flowing of rural factors such as land, population and capital, pushed urban factors into the countryside, and expanded the scope, intensity and direction of rural factor flows. In addition, there were always mutual feedback of elements between rural factor flow networks and external urban environment. Since the 21st century, with the comprehensive promotion of the rural revitalization strategy, urban capital, population, technology and public services had accelerated the reversed flowing into the countryside, especially to the central villages and township, further promoting the formation and development of the multi-center structure of the factor flow networks in the rural areas.

In addition, the influence of technology and energy on rural factor flow networks was also remarkable [60–62]. Technological advancements could directly change the flowing speed and path of population, capital and transportation factors and promote the transformation of rural functions, thereby affecting the operation process of rural factor flow networks. For example, Internet technology created digital economy and virtual social interaction, which accelerated the trans-local flowing of rural population, capital and information. In particular, the development of online shopping enhanced the connection between general villages and external regions. Moreover, to cope with climate change and environmental pollution, the Chinese government is actively pushing rural energy consumption from coal to natural gas and electricity. However, for rural areas with residential dispersion and low population density, the cost to use and maintain of natural gas transported by pipeline is relatively high, which promotes the flowing of rural population and capital to the township and central villages with complete infrastructure and low energy use-cost, and promotes the polarized development of township and central villages, thus reconstructing the rural factor flow networks.

### Town-village symbiotic theory: A new type of town-village development relationship

**Fig 6:**
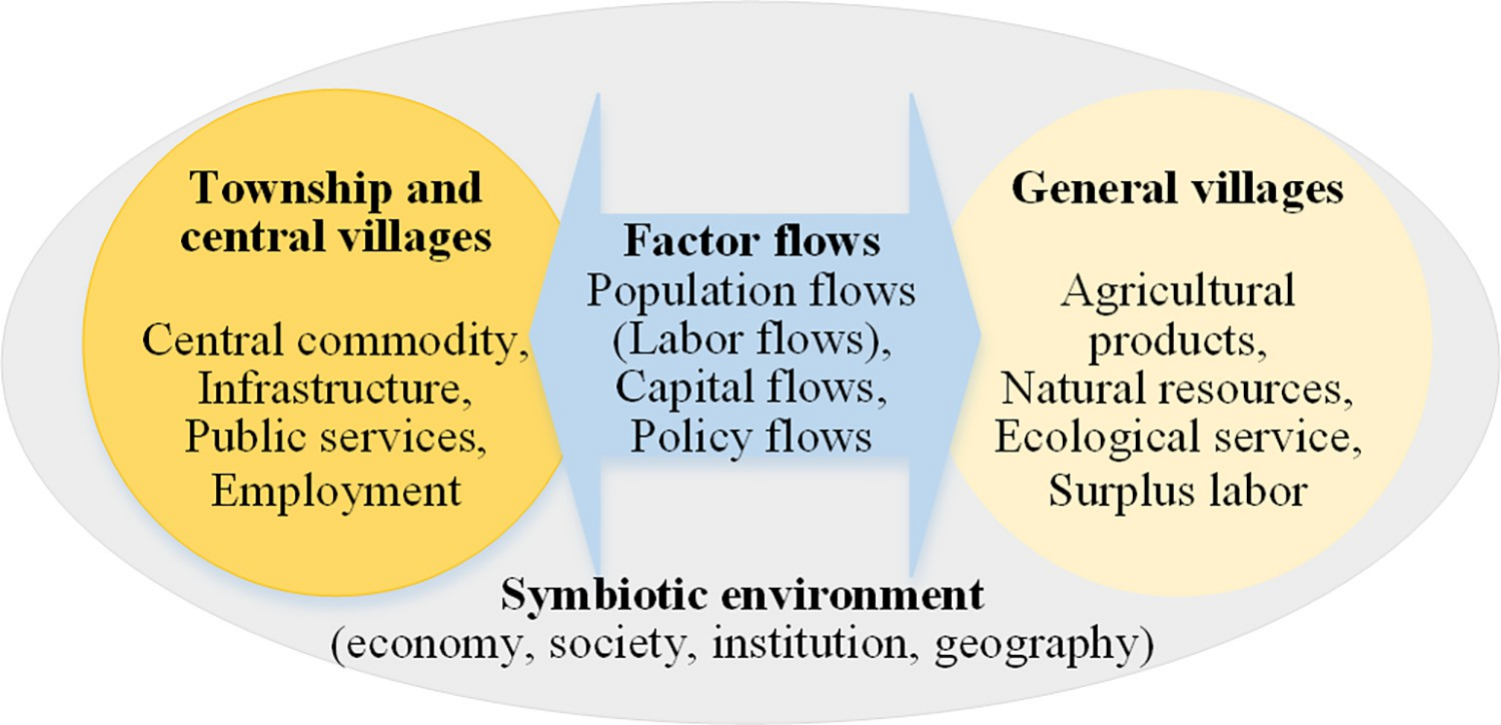
Theoretical model of town-village symbiotic network.

Rural endowments, factor flows and regional environment played different roles in the operating process of the rural factor flow networks. Central villages, township and other villages interacted closely, forming a new integrated development relationship. Based on the theory of symbiosis [63–65], this paper established a theoretical model of town-village symbiotic system containing three conceptual elements: town and village symbiotic unit, factor flows, symbiotic environment (Fig 6). Thereinto, villages and township were the basic units of production and cooperation in the symbiotic system. Central villages and township had higher-grade goods, critical infrastructure, employment positions, and public services; comparatively, general villages had agricultural and sideline products, land resources, water resources, surplus labor, and ecological conservation services. Factor flows refer to the process of bidirectional flowing and interaction between villages and towns in population, capital, transportation, policy, organization, and other factors. The symbiotic environment is an external condition for the development of symbiotic units in rural areas, including economic environments (industrial structure, leading industry and innovation), social environments (social organization and culture), institutional environments (institution, policy and local management), and geographical environments (geology, landscape, climate, soil, hydrology and vegetation).

The theoretical connotation is that, in the regional symbiotic environment, central villages and township constitute a new type of town-village symbiotic development network through the close interweaving of factor flows and spatial interaction. The theory takes into account the endowments of central villages, township and general villages, as well as the sustainable development goals on food production, economic productivity and sharing of social services, which will be expected to be the main theoretical basis to guide the rural revitalization in Chinese traditional agricultural areas.

### Limitations and prospect

The paper was the first to use the fine-grained flows data to analyze the operational characteristics of population flow network, capital flow network and policy flow network and the relations of multi-factor flows in rural areas. However, this paper did not take the rural information flows into account, nor the complex relationship between the rural factor flow networks and the network outside the region, which would be further studied in the future work. In addition, this paper initially revealed the disunity and competitive-cooperative relationship between the economic center and the administrative center. Compared with the single-center structure, the multi-center structure of flow networks was more conducive to the accessibility of ordinary villages to obtain employment, commodities and public services, and also helped to improve the stability of network. Then, what is the changing threshold and theoretical model of the competitive-cooperative relationship between economic center and administrative center in rural areas, and whether it is affected by scale? These problems need to be deeply explored in the study of rural revitalization.

## Conclusions

Taking a typical industrial town in China as an example, the study explored the operating characteristics of rural factor flow networks from three dimensions, including population flow network, capital flow network and policy flow network, and the relationship of multi-factor flows. On one hand, population flows, capital flows and policy flows had increased significantly in rural areas. Thereinto, population flows, especially labor flows, mainly ran into central towns and industrial cluster villages, so did capital inflows and outflows, and policy flows ran around the township. The villages with dense population and capital flows formed the "central villages", which had exceeded the township in the two flow networks. In short, a multi-center structure network with the separation of economic center and administrative center had been formed in rural areas. On the other hand, there was a close interaction between these factor flows. specifically, Policy flows and capital flows played a guiding role in population flows, so did the policy flows on the capital flows, as a result, there was both guidance and competition between the two flows. Meanwhile, the population flows and the capital flows could reinforce each other.

This paper discussed the main elements influencing the formation of the multi-center structure of the rural factor flow networks, including rural endowments, regional environment, energy flows and technological advancements, and summarized the advantages of this operating characteristics. Furthermore, the theoretical model of town-village symbiotic network was constructed. That is, in the regional symbiotic environment, central villages/township and general villages constituted a new type of town-village symbiotic development network through the two-way flowing of factors, which taken into account the multiple functions and diversified development goals of rural areas, and would provide theoretical and methodological reference for rural revitalization in traditional agricultural areas in China.

## Supporting information

S1 Table Descriptions and characteristics of several administrative terms. (DOCX)
