# Supplementary material for: Operating characteristics of the factor flow networks in rural areas: A case study of a typical industrial town in China
Source: PLoS One. 2023 Mar 16;18(3):e0283232. doi: 10.1371/journal.pone.0283232 (PMC10019707; doi:10.1371/journal.pone.0283232)
Supplement: S1 Table — (DOCX) [file pone.0283232.s001.docx]

**S1 Table. Descriptions and characteristics of several administrative terms.**

| **Terms** | **Descriptions** | **Case** | | |
| --- | --- | --- | --- | --- |
|  |  | **Region** | **Jurisdiction** | **Areas (km^2^)** |
| **Province** | The highest level of administrative region in China. | Hebei  Province | 97 Counties. | 188,800 |
| **County** | The administrative division under the jurisdiction of Province. | Ningjin  County | 14 Towns. | 1,111 |
| **Town** | The administrative division under the jurisdiction of County. | Jiajiakou  Town | 18 Villages. | 94 |
| **Village** | The smallest administrative unit below the Town. | 18 villages | - | 0.73~11.13  Average:4.80 |
| **Township** | The village where the town government resides | Jiajiakou village/township | - | 7.02 |
